# Is Social Media a New Type of Social Support? Social Media Use in Spain during the COVID-19 Pandemic: A Mixed Methods Study

**DOI:** 10.3390/ijerph19073952

**Published:** 2022-03-26

**Authors:** Aviana O. Rosen, Ashley L. Holmes, Nekane Balluerka, Maria Dolores Hidalgo, Arantxa Gorostiaga, Juana Gómez-Benito, Tania B. Huedo-Medina

**Affiliations:** 1 Department of Allied Health Sciences, University of Connecticut, Storrs, CT 06269, USA; aviana.rosen@uconn.edu (A.O.R.); ashley.holmes@uconn.edu (A.L.H.); 2 Department of Clinical and Health Psychology, and Research Methods, Faculty of Psychology, University of the Basque Country UPV/EHU, 20018 Donostia, Spain; nekane.balluerka@ehu.eus (N.B.); arantxa.gorostiaga@ehu.es (A.G.); 3 Department of Basic Psychology and Methodology, Faculty of Psychology, University of Murcia, 30100 Murcia, Spain; mdhidalg@um.es; 4 Group on Measurement Invariance and Analysis of Change (GEIMAC), Institute of Neuroscience, University of Barcelona, 08035 Barcelona, Spain; juanagomez@ub.edu; 5 Department of Social Psychology and Quantitative Psychology, Faculty of Psychology, University of Barcelona, 08035 Barcelona, Spain

**Keywords:** COVID-19 pandemic, social media, social support, qualitative research, survey research, health promotion, interpersonal communication

## Abstract

This study examines Spanish adults’ social media use during the COVID-19 pandemic using mixed-methods to assess and understand frequency, context, and changes in social media use during two critical time points in Spain. We conducted semi-structured interviews in April 2020, and two waves of surveys (April 2020, April 2021) among Spanish adults. We coded and analyzed qualitative data related to social media use during the first lockdown period in Spain using Dedoose software; and ran descriptive statistics and chi-square tests to assess changes in social media use over the two survey waves related to perceived social support and loneliness. Participants ranged in age from 18–92 and were representative of the Spanish population’s sociodemographics. Interview data show that WhatsApp was most commonly used, and that social media allowed for social support and engaging in healthy behaviors. Survey data show that women and individuals aged 18–34 had the greatest increases in social media use. Statistically significant associations were found between social support and loneliness with social media use. Our results show that promoting social media use as an emotional resource for social support in times of crisis or isolation can minimize loneliness and can be a beneficial tool for general worldwide crises.

## 1. Introduction

Since the beginning of the COVID-19 pandemic in 2020, dramatic increases in social media use have been widely cited from countries across the globe, with 4.2 billion users of social media almost a year into the pandemic in January 2021, compared to 3.8 billion the year before [1,2,3,4,5,6,7]. As of October 2021, this number has increased to 4.55 billion social media users, exhibiting a 9.9% growth [8]. Further, Western Europe and North America have the highest social media saturation rates in the world, and Spain in particular has been cited as seeing massive increases in media consumption for news, instant messaging applications, and social media platforms since the start of the pandemic [6,9,10].

At the start of the pandemic when there were so many uncertainties about SARS-CoV-2, Spain was one of the hardest hit countries in Europe, with extremely strict government lockdowns and restrictions of social gatherings, forcing its population into social isolation [11]. Research has found that since the start of the pandemic, social distancing regulations contributed to increases in social media consumption, and that many engaged in intensive social media use to stay informed about the unfolding COVID-19 [12]. A recent report found that the number of social media users in Spain increased by 28% between 2020 and 2021 [8]. Another study that examined the relationship between media consumption and psychological well-being among Spanish adults found that there was a significant increase in mean minutes spent per day on social media during lockdown compared to before [6]. In fact, more than half of participants in this study reported frequent use of social media as an information source, and the increased social media use was significantly positively related to anxiety.

In addition to increased social media use in Spain, the digital communication platform WhatsApp has seen an 83% growth rate during the pandemic, making Spain the country with the highest growth in the world [13]. Within this growth, WhatsApp has transitioned from being a personal messaging app, to more of a social media mechanism, with use as a platform to share news, media, photos and videos, and communicate via message and video call with groups and individuals [14]. As of October 2021, WhatsApp was announced the third top used social media platform in the world, behind YouTube and Facebook, and was rated the ‘favorite’ social media platform among internet users aged 16–64 [8].

While there is no doubt that social media use has increased during COVID-19, the literature is mixed on whether this has positively or negatively affected health outcomes. Generally, social media and smartphone use have been associated with negative effects on mental health, physical health, health behaviors, social isolation, happiness, and mood [15,16,17,18,19,20,21,22,23,24,25]. Other research on social media and health-related social media interventions have found that when used with proper guidance, social media use can have great implications for behavior change including increased interaction with others and enhanced social and emotional support [22,25,26,27,28,29,30]. During the COVID-19 pandemic, several studies have found that social media had negative effects on health and well-being. For example, a study of social media use on mental health, quality of life, well-being, and loneliness found that among adults in the United States, United Kingdom, Norway, and Australia, those who reported high frequent use of social media in the first weeks of the pandemic experienced poorer mental and psychological health [31]. Another study among adults in China found that weekly social media use significantly increased during the first COVID-19 lockdown period compared to before, and females reported greater increases [32].

Similarly, research among Spanish adults during the lockdown in April 2020 found that those who increased their use of social networking sites during the lockdown had a negative correlation with their level of happiness [6]. Further, those who reported greater well-being also reported spending less time on social networking sites, and those who engaged in more use of these sites were less happy than those who did so modestly, and had lower general and social well-being [6]. Contrarily, a study of psychiatric outpatients in Spain found that those who used social media more frequently during the lockdown period engaged in more physical activity [33], and that those who reported higher anxiety symptoms were more active in social networking apps (e.g., Facebook, Instagram, Twitter) and less active in communication apps (e.g., WhatsApp, telegram) [34].

Other research has found that if smartphone and social media use is attributed to seeking social support, it may help guard against the negative effects of the deprivation of in-person contact and other pandemic-related stress [33]. One review studying the association of social support and health with Facebook use found that when individuals used Facebook for social support, they experienced better mental and physical health outcomes [35]. These data suggest that if social media is used to engage in meaningful social connections, especially during a time of forced social isolation and stress, the social networking use can be beneficial to well-being [6,33,36].

While many studies have cited increases in social media use during the pandemic [2,3,4,5,6,7,8,12,13,14], few studies have assessed the reasons and context behind these increases, or changes in social media use over time during the pandemic, particularly among Spanish adults during the changing COVID-19 climate and regulations. With the data mixed on whether this social media use is harmful to physical and mental health and well-being may largely depend on the motivations and reasons for an individual’s use. Therefore, the purpose of the present study is to provide insight into the drastic increases in social media use during the COVID-19 pandemic among Spanish adults, using mixed-methods to assess and understand frequency, context, and changes in social media use during two critical time points of the pandemic in Spain. We conducted semi-structured interviews in April 2020 during the time that Spain was one of the first countries outside of Asia to be strongly hit by the pandemic [37], and quantitative surveys in April 2020 and April 2021 among Spanish adults. Based on the reviewed literature, we hypothesized that social media use will increase throughout the course of the pandemic, and that social media communication apps will be commonly used for social support and to reduce loneliness among participants.

## 2. Materials and Methods

### 2.1. Qualitative Methods

For this project, in order to thoroughly obtain the nuances, perceptions, and points of views from the narratives of Spanish adults during the first COVID-19 lockdown period, we conducted semi-structured in-depth interviews. Following guidelines outlined by Weiss (1995) [38], we developed interview questions based on our study goals and concepts critical to the study and used prior literature on epidemic and pandemic population effects to ensure we included questions that would address the necessary aspects. We developed key questions to ask during the interviews to guide the interviewer on the primary areas to explore but utilized a semi-structured format allowing the interviewer to flexibly pursue any unforeseen concepts that arose in the interview [39]. We used an inductive approach to data coding to develop codes and themes related to social media use from the data, which is most often identified as an aspect of Grounded Theory [40,41,42].

#### 2.1.1. Recruitment

In collaboration with researchers from different Spanish universities, researchers at the University of the Basque Country (Spain) led a mixed-methods study on the psychological effects of the pandemic and government-imposed lockdown on the Spanish adult population. An initial report of the data has been published [43]. Recruitment was executed by the Strategic Market Research Solutions (Soluciones Estratégicas de Investigación de Mercados; SEIM) Company, a reputable research and recruitment organization based in Madrid, Spain. SEIM used a tiered recruitment system to contact adults 18 years and older in Spain through their broad network of records. This recruitment was based on theoretically relevant criteria for the purpose of the study, including level of vulnerability to the SARS-CoV-2 virus (occupational, health, exposure, age), geographical area (representing the difference incidence rates in the regions of Spain), and personal living situation (living with dependents such as children, elderly, or individuals with chronic illness). In the first tier of recruitment, individuals who were in a sensitive or vulnerable pandemic situation (e.g., vulnerable due to comorbidities, live with vulnerable individuals, consistent exposures, live with exposed individuals) were contacted as they are typically the most difficult to recruit. In the second and third tiers, lower-risk individuals were contacted. The individuals contacted for recruitment were balanced in gender, age, geographic regions of Spain, occupational situations, family and housing situations, and relationships to COVID-19 (e.g., diagnosed, not diagnosed, loved ones diagnosed). SEIM contacted individuals and informed them of the study objectives, characteristics, and confidentiality agreements with the European General Data Protection Regulation and informed them about the ethics approval by the Committee for Research with Human Subjects at the University of the Basque Country. If participants wished to continue, a team member from SEIM contacted them to inform about study objectives and the general content of the interview. Participants were notified that their data would be confidential, and those who consented to participation provided verbal consent recorded on audio. Once consent was obtained, a date and time for conducting the interview at the participant’s convenience was arranged.

#### 2.1.2. Interview Question Development

The scientific literature on the effects of COVID-19 and prior pandemics and epidemics was thoroughly searched to find inspiration for evaluating the personal and psychological consequences of the pandemic that would inform the semi-structured interview questions. Additionally, several members of the research team are experts in the psychological impacts of traumatic situations (e.g., disasters, loss, sudden illness or death, extreme stress) and were able to inform questions that may help address our research questions.

Each interview question sought to elicit details on Spanish adults’ experience during the lockdown in Spain regarding psychological, cognitive, behavioral, and emotional effects derived from the pandemic and lockdown regulations. We also sought to describe any contextual variables that may have influenced these effects including housing, economics, social support, COVID-19 exposure or diagnosis; and be able to describe any changes in these effects from before to during the pandemic. The interview questions were sorted by area into nine subject blocks including employment, housing characteristics, perceptions of COVID-19 regulations, basic habits during lockdown, emotional impact, coping strategies, and more; outlined in Table A1. Questions that commonly elicited responses including aspects of social media or mass media include those related to basic habits, effects of the lockdown on family and friend relationships, emotional impacts, and coping strategies.

#### 2.1.3. Semi-Structured Interviews

Semi-structured interviews were conducted by four Spanish researchers with doctorate degrees in psychology and expertise in conducting qualitative interviews. The interviewers were trained on interview protocol, probing techniques, and crisis or mental health management in instances of detecting clinical indicators of severe or harmful distress in the participant. The interviews were conducted by phone through WhatsApp video calls in the second week of April 2020 (the fifth week of the first lockdown in Spain) and were recorded with the consent of the participants. Interviews were conducted by conventional phone call in the event of poor internet connection, or if participants did not feel comfortable showing their face on camera.

Interviews were conducted until saturation was reached. Saturation is the “gold standard” for the point at which it is acceptable to discontinue data collection in qualitative research; when no additional data are being found, and no new codes are occurring in the data [40,44,45]. With this aim, the four interviewers and the research team met daily to evaluate the interviews and examine the new codes that emerged until no new themes emerged. The interview recordings and transcriptions were stored anonymously on a password protected storage drive at the University of the Basque Country, in compliance with the ethics committee for Research with Human Subjects at the University.

#### 2.1.4. Data Analysis

Using Dedoose software [46], a researcher with qualitative experience (A.O.R.) coded the interview transcripts. As outlined above, we used inductive coding practices to search for meaning in recurring concepts and themes in the interviews, without applying predetermined theoretical frames [40]. With inductive coding, we were able to draw from the data and allow non-predetermined categories to emerge using Thematic Analysis [47,48,49]. Coded data included mentions of various social media platforms (e.g., Facebook, Twitter, Instagram) in addition to mentions of WhatsApp, YouTube, Skype, and Zoom when related to personal life. We coded mentions of video calls, although we did not code data regarding strictly telephone calls or work-related media use for meetings. A total of 41 interviews were conducted, although due to technological issues with the virtual call, one interview was incomplete and thus removed from the dataset. Additionally, two interviews had no mention of any social or mass media use, and thus were also removed from the dataset.

### 2.2. Quantitative Methods

We collected data in two waves during key moments in the COVID-19 pandemic in Spain. The first, in April 2020, Spain was the European country with the highest number of confirmed COVID-19 cases. The Spanish government had declared a State of Emergency with 202,990 cases of COVID-19, and 22,524 COVID-19-related deaths. The second, April 2021, several autonomous communities in Spain were coming out of a second lockdown and vaccines were starting to be distributed.

#### 2.2.1. Recruitment

The company Netquest recruited a representative sample of Spanish adults over 18 years old. For sampling, the key sociodemographic variables of the population were considered as quotas, being representative of gender, socioeconomic status, autonomous community (i.e., region) of Spain, and age. Netquest sent recruitment invitations to 16,205 individuals; 10,059 were accepted, with a 62% recruitment rate. Of the 10,059 participants that consented to participate, 271 were excluded for various quality filters, 42 did not follow-up thus were excluded, 45 were excluded as the quota for their given autonomous community had already been met, 2297 did not meet the survey deadline, and 615 were incomplete. In Wave 1 (April 2020), a total of 6789 participants completed the survey. For Wave 2, Netquest resurveyed 3500 of the same participants who had completed the Wave 1 survey.

#### 2.2.2. Survey Measures

The survey questionnaire for both waves consisted of COVID-19-related questions assessing changes in lifestyle, mental health, psychological effects of COVID-19, living situation, COVID-19 risks and vulnerabilities, sociodemographic characteristics, among others. For this project, we focused on the questions related to social media use and social support. In Wave 1, social media use was assessed with the question, ‘Compared to your life before the lockdown, how has your social media use changed during lockdown?’ with responses ranging from 1 (has decreased a lot) to 5 (has increased a lot). For data presentation purposes, we combined Options 1 and 2 to represent a decrease in social media use, and 4 and 5 to represent an increase in social media use. To measure social support, we used perceived levels of help and support as a proxy for perceived social support. Participants were asked to identify on a scale of 1 (no help) to 10 (a lot of help) how much help they believed they would have if the following situations of need presented themselves: physical or medical problems, psychological problems, need for social support (e.g., chatting, sharing feelings), need to buy food or supplies, housing infrastructure problems (e.g., electric, water cut off), and need to run errands. We combined options 0–3 to represent little to no help, 4–6 for some help, and 7–10 for a lot of help. Finally, we measured feelings of loneliness by asking participants how their feelings of loneliness changed during lockdown compared to before the pandemic. Responses were collected and combined in the same manner as changes in social media use.

Wave 2 used a similar social media measure and scale, but built on Wave 1′s question, and asked, ‘Since last summer, how has your social media use changed?’. The proxy for social support in Wave 2 was similarly related to need for resources and whether participants had access to help by asking, ‘In the last 30 days, have you had difficulty accessing important daily resources (e.g., sanitary products, food, clothes, water, house supplies, medication)’ with answer choices of yes or no. Participants who answered yes were then asked to evaluate how stressful these difficulties were on a scale of 1 (not at all stressful) to 5 (extremely stressful). Responses were recategorized to represent not very stressful, somewhat stressful, and very stressful. We hypothesized that participants who had less difficulties may have had more social support with help getting these supplies, and those who did have difficulties obtaining supplies would report higher increases in social media use if they felt more stress, thus leveraging social media to communicate with others for help.

#### 2.2.3. Statistical Analyses

IBM SPSS Statistics Version 27 was used to run all statistics [50]. Descriptive statistics were run for demographic variables; and Chi-squared tests were used to test possible associations between categorical demographic variables and the two time points (categorical ordinal variables) [51]. We tested the null hypothesis of independence between the two waves of data collection and each of the following variables: social media use and perceived levels of help/social support (Wave 1), loneliness (Wave 1), age and gender (Wave 1), and social media use and presence and level of stress accessing important daily resources (Wave 2). Associations were considered significant at *p* < 0.05.

## 3. Results

### 3.1. Qualitative Results

The mean age of the 40 participants is 43.6 (SD = 14.1). Slightly more than half are female (*n* = 21, 52.5%), between the ages of 36–55 (*n* = 21, 52.5%), and hold a bachelor’s degree or higher (*n* = 32, 80%) (Table 1). The participants were representative of the 13 autonomous communities of Spain, and for their employment status, most were on paid temporary leave from work (*n* = 11, 27.5%) or working from home (*n* = 8, 20%). Most participants (*n* = 16, 40%) live with a partner, 9 (22.5%) live with young children, 6 (15%) live with older people, 5 (12.5%) live alone, 2 (5%) live with friends, and the remainder reported having mental health or addiction problems (*n* = 2, 5%). In terms of participants’ relation to COVID-19, 22 (55%) participants reported not being affected at all by the virus, meaning that they were not infected or in close contact to someone infected, nor were any of their friends or family. Seven (17.5%) participants had been infected with COVID-19, 5 (12.5%) had a close friend or family member test positive, 6 (15%) lived with individuals who are vulnerable or at-risk for exposure, and 5 (12.5%) reported having been in close proximity with someone who had tested positive.

Throughout the interviews, there were seven mentions of social media use in general. In terms of specific application use, there were 7 mentions of Instagram, 4 of Facebook, 1 of Twitter, and 4 of YouTube. Five participants said that they used social media more during the pandemic than they did before COVID-19, and one participant said they use less social media than before the pandemic. Three participants made a point to say that they were spending ‘a lot of time’ on social media. Reports of WhatsApp use were much more common, as there were 42 specific mentions of WhatsApp use across the interviews, and 12 mentions of using WhatsApp to communicate with groups of friends, family, or neighbors. There were 9 co-occurring codes where participants emphasized spending a lot of time on the phone using WhatsApp in particular.

“Sometimes we send humorous things, and in these situations it brings a smile and it makes you happy, I believe it’s positive, WhatsApp taking away [the stress and suffering] a little…I believe [WhatsApp] for me is important and for a lot of people too.”

—Male, 73, Basque Country

Video calls were the most coded concept across the interviews, with 80 total mentions of video call use for personal reasons. There were 20 coded co-occurrences of WhatsApp with video calls, while four participants specifically mentioned the use of Zoom, and four participants specifically mentioned the use of Skype. Ten participants identified that they had changes in their daily routine related to video calls since the pandemic started. There were 13 co-occurring codes where participants emphasized spending ‘a lot of time’ on the phone specifically for video calls, and 9 co-occurrences where participants said they had more contact with others and more video calls during the pandemic compared to before. Finally, there were 12 co-occurrences for video calls and communicating for the purpose of social support.

“We have a lot of friends in Italy who have been [in lockdown] since February and so we knew the gravity of this and we confined ourselves at home 10 days earlier. Of course, social media has served as a way to care for each other. I call my mother very often, but now I send her things on YouTube everyday so that she exercises, cooks, she prepares breakfast and I prepare dinner. It’s there to enhance affections and to connect you to the people who you aren’t able to see.”

—Female, aged 36–55 (not specified), Catalonia

Several themes related to media use were identified during the inductive coding process including change in routine, social support, being face-to-face or seeing faces, news in the media, media use for hobbies and healthier living, virtual hangouts or events, and media use for pandemic relief. Table 2 provides detailed analyses of these themes.

### 3.2. Quantitative Results

In Wave 1, the 6789 participants ranged in age from 18–92 (mean = 45.8, SD = 14.5). Participants were about half female (*n* = 3534, 52.2%), mostly single (*n* = 2715, 40%) or married (*n* = 3251, 47.9%), and highly educated with an advanced degree (*n* = 1833, 27%). The majority of participants worked in the private sector (*n* = 2398, 35.3%), and maintained their employment status when the COVID-19 pandemic started (*n* = 2994, 44.1%). Additionally, about 30% (*n* = 2251) were considered to be part of a COVID-19 risk group (e.g., elderly, immunosuppressed or chronic illness, pregnant). The 3500 Participants in Wave 2 who were a subset of participants from Wave 1 had similar sociodemographic characteristics and ranged in age from 19–88 (mean = 48.2, SD = 14.2). Table 3 contains complete demographic characteristics of the Wave 1 and Wave 2 samples.

Table 4 presents the changes of social media use from Wave 1 to Wave 2. In Wave 1, the majority of participants reported a small or large increase in their social media use since before the pandemic (*n* = 4675, 68.9%), and about 25% (*n* = 1735) reported no change in their social media use. In April 2021, participants were asked if their social media use had changed since the previous summer (2020). Among participants, about 47% (*n* = 1643) reported no change since the previous summer, and about 43% (*n* = 1487) reported a further increase in social media use.

Associations between social media use and sex and age in Wave 1 were statistically significant (Table 5). High percentages of both males and females reported increases in social media use during the lockdown period compared to before the pandemic, although slightly more women reported increases than men (75.5%, *n* = 2612; 66%, *n* = 2063, respectively). A similar pattern was seen across age categories as well, with the majority of participants reporting increased social media use, although slightly more among ages 18–34 (76%, *n* = 1352) and 35–60 (71.2%, *n* = 2440), compared to 64% (*n* = 883) of participants over 61 years old.

Associations from Wave 1 (Table 6) show statistically significant associations between increases in social media use during April 2020 lockdown compared to pre-pandemic and all proxies for social support. Particularly for participants who felt that they would have a lot of help if they needed social support (e.g., to chat with others, share feelings with others), almost half (*n* = 3195, 47.1%) had increased social media use (χ2 (60, *n* = 6789) = 187.80, *p* < 0.05); similarly, for those who felt they would have a lot of help if needed for buying food and supplies (*n* = 3250, 47.9%; χ2 (60, *n* = 6789) = 118.59, *p* < 0.05). Additionally, there was a significant association between loneliness and changes in social media use (χ2 (30, *n* = 6789) = 288.04, *p* < 0.05), and a large percentage of participants who reported increased loneliness, also increased their social media use (28.5%, *n* = 1938).

Statistically Significant associations were found in Wave 2 as well (Table 7). Similar to Wave 1, we used difficulty in accessing important daily supplies as a proxy for social support, hypothesizing that participants with less difficulties may have had more social support to help them get these supplies, and if they did have difficulties getting supplies and found them more stressful would report increased social media use, suggesting they turned to social media for support in getting these supplies. Of participants who reported difficulties in obtaining supplies (*n* = 722, 20.6%), about half of them reported increases in social media use since the prior summer (*n* = 355, 49.2%), and 297 reported no change in their social media use since the prior summer (41%). Among the 722 participants who reported difficulties, the highest percentage (19.9%, *n* = 144) reported them as being very stressful and increased their social media use from the prior summer or reported them as being somewhat stressful and increased their social media use (16.9%, *n* = 122).

## 4. Discussion

Results from both waves of our survey data show that social media use dramatically increased among adults in Spain during the first lockdown period in April 2020 across sex and age, and mostly either remained the same or further increased in April 2021, a year into the pandemic. Our qualitative data provides some context to these numbers, suggesting that Spanish adults were leveraging social media during the first lockdown period to increase social support via virtual calls or WhatsApp, increasing physical activity or learning new recipes with Facebook or Instagram accounts, some to view the news, and others just standard social media use (e.g., relaxation, look at memes, relief from the pandemic).

Interestingly, we found that several individuals used social media as an innovative way to get healthier and learn new skills in lockdown. Instagram and YouTube exercise videos and fitness accounts were brought up by several participants as ways to stay healthy and active while COVID-19 regulations kept gyms and fitness centers closed. Some other studies during the pandemic had similar findings. A study conducted by Aksoy et al. (2021) found that among adults in Turkey, social media influence was positively related to COVID-19 fear, but family influence and social media influence were positively related to health consciousness, with participants reporting engagement in more hobbies, cooking, and physical fitness [52]. Another study by Gupta et al. (2021) found that the reasons for increased physical activity during lockdown among medical students in India were learning new exercises online; using apps for exercise; and engaging in online yoga, dance classes, and motivational videos [53]. Finally, a study conducted surveys and focus groups of adults in the United Kingdom and found that during the COVID-19 lockdown periods, many used social media to self-manage physical activity, diet, and quality of life through accessing workouts, new recipes, and new opportunities to interact virtually with peers and family members [19]. Our results are consistent with these studies and suggest that with proper direction, social media can be used to promote healthy behaviors and benefit health and well-being. Social media is a particularly critical tool for promoting health behaviors and social interaction during times of crisis, especially when fitness centers are shut down.

Our results regarding social support share similarities and differences with other studies conducted during the April 2020 lockdown in Europe. A study by Arpino et al. (2021) conducted in April 2020 during the first lockdown assessed adults in Spain, Italy, and France, countries who also saw extremely high incidence rates of COVID-19 and related deaths [54]. This study found that a large portion of older adults aged 50 and over increased their use of video calls, instant messaging, and social media; and that these virtual contacts may have been crucial in limiting the negative effects of isolation and reduced physical contact with loved ones [55]. Prior research has found that generally, social contacts decline with age [56], although during the first lockdown, adults aged 50 and older saw a 45% increase in video calls, and a 54% increase in instant messages [54]. Similar to our results, we saw a 64% increase in social media use among older adults aged 61 and older, and high increases among younger adults as well. Important to note, Spain’s social media increases were higher than those found in Italy or France. For example, Spain saw a 52% increase in video calls, and 67% increase in instant messaging, much higher than increases in France (36%, 38%) or Italy (48%, 57%) [54].

Although our surveys did not directly assess which social media platforms were used the most, our qualitative data suggests that WhatsApp was the most commonly utilized as it was mentioned the most frequently of all social media platforms. This finding is consistent with our hypothesis and other data regarding WhatsApp use globally and in Spain during the pandemic. Another report found that almost half (48%) of all global internet users aged 16–64 reported their main reason for using social media was to stay in touch with family and friends; followed by filling up spare time, reading news stories, and finding funny or entertaining content [8]. These results are consistent with our qualitative findings, and show that maintaining social support through social media and communication platforms has become a main driver for social media use. In our qualitative data, more participants cited increased social media use as a way to receive social support from friends and family than for general use or for news. It is clear that participants utilized the tools that social media provides us with during this difficult time of isolation, to maintain their relationships and continue to receive the social support that was needed. Both our qualitative and quantitative data suggest that this contributed to participants feeling less alone, and more supported.

Further, our survey data shows that participants who reported increased feelings of loneliness during the first lockdown period in Spain also significantly increased their social media use. Our data are consistent with the findings that social media is a favored outlet for social support, particularly during times of mandated social isolation [6,33,35,36]. Our proxies for social support in Wave 1 including having physical or psychological problems, needing social support, needing food or supplies, among others, also found significant associations with reported increases in social media use. Our data shows that individuals who perceived they would have a lot of help with any of these issues reported increases in social media use. Based on the social support and social media use literature during the pandemic and our qualitative findings regarding WhatsApp use and social support, we believe this could be attributed to individuals feeling more supported via WhatsApp, and feeling as though they would be able to reach out to a family member or friend for help with any of these problems or needs.

Similarly, our survey data from Wave 2 have similar implications for social support and social media use. Participants who reported difficulties accessing important daily supplies in April 2021 and appraised these difficulties as somewhat or very stressful reported either no change, or increases in their social media use since the previous summer (2020). Given that the majority of participants reported an increase in social media use in April 2020, our data suggests that the participants in Wave 2 who reported no change in social media use since the prior summer still had higher than normal engagement in social media use. Thus, using difficulties in accessing supplies and the stress appraisal for these difficulties, our data from Wave 2 suggests that the high engagement in social media use among those who report feeling somewhat or very stressed may be to help reduce their stress in accessing these supplies by asking for help and support from others.

A pandemic report found that Spain was the top third European country for increases in social network activity during the beginning of the COVID-19 pandemic [7], and our data validates these increases. Early reports found these social media increases as being harmful to mental and physical health [6,31,32,34], although with recent data and our data suggesting there were several benefits to these increases such as social support, it is possible that without initial qualitative data to assess the reasons and implications of the social media use increases, the negative results originally found were confounded by experiences of stress, anxiety, depression, helplessness, and loneliness that were experienced by so many during the pandemic. Thus, while in general, overuse of social media can potentially be harmful and cause lower mental health, our data provides evidence that in a time of crisis and isolation, social media use can be leveraged for health benefits and needed social support.

While our paper is the first to qualitatively assess social media use during the first lockdown period in Spain at the beginning of the COVID-19 pandemic, it does have limitations. First, as our data were collected at the beginning of the pandemic, it is possible that some aspects of our results may have changed during the course of the pandemic. For example, some participants noted having “video call fatigue” or general fatigue of social media use just 10 days into the lockdown period, that if reassessed months later, many more may have felt this way [57,58]. Second, while our recruitment process was extremely thorough through NetQuest and sought to recruit a sample that represented the various sociodemographic variables of Spanish adults, our sample needed internet access since they were recruited by email, thus Spanish adults who do not use email, or do not have regular internet access may have provided very different results about social media use during the pandemic lockdown. Further, we made an effort to recruit frontline workers and other individuals with jobs that place them at risk for COVID-19 but were not successful in this endeavor. Future research would benefit from interviewing individuals in these at-risk occupations. Finally, we did not specifically ask about social media use in our interviews, how individuals accessed their news, how often they used social media and what platforms; rather, we found that it was a recurring theme throughout the interviews through other questions asked and provided interesting data that would be beneficial to report. If we had directly assessed these aspects of social media use we may have uncovered more data.

Despite these limitations, our research has notable strengths. Using a mixed-methods approach, we were able to not only provide statistics on the increases in social media use among Spanish adults but were also able to provide detailed context for what exactly individuals were using social media for during the time of the first lockdown. Additionally, we were able to collect data longitudinally, and are able to compare results among the survey sample over time, at different key timepoints of the pandemic.

## 5. Conclusions

Overall, our quantitative data provide evidence of substantial increases in social media use and how increases may be associated with feelings of social support. Our semi-structured interviews provide detailed context to how Spanish adults were leveraging social media during the first lockdown for social support and other health benefits, and why their usage increased so dramatically. We found that most individuals were using social media for positive health outcomes including seeking social support, learning new hobbies, finding healthy recipes, and for exercising. Our results suggest that maximizing the potential of social media as an emotional resource to maintain or increase social support during a time of forced isolation and uncertainty may be beneficial for mental and physical health outcomes, and future research should test campaigns that promote social media as a means of social support in stressful or uncertain times.

## Figures and Tables

**Table 1:** Qualitative Participant Demographics (*n* = 40).

Variable	*n* (%)
Gender	
Female	21 (52.5)
Age Category	
18–35	13 (32.5)
36–55	21 (52.5)
>55	6 (15)
Employment	
Lost job	5 (12.5)
Not working	6 (15)
On ERTE *	11 (27.5)
Retired	6 (15)
Student	4 (10)
Working from home	8 (20)
Education	
Primary studies	2 (5)
FP1	2 (5)
Bachelor’s or FP2	10 (25)
University **	14 (35)
Postgraduate	6 (15)
Doctorate	2 (5)
Autonomous Community	
Catalonia	5 (12.5)
Aragon	2 (5)
Baleares	1 (2.5)
Basque Country	3 (7.5)
Castilla-La Mancha	2 (5)
Castilla-Leon	2 (5)
Andalucía	3 (7.5)
Extremadura	4 (10)
Galicia	3 (7.5)
La Rioja	4 (10)
Madrid	6 (15)
Navarra	2 (5)
Valencia	3 (7.5)

* ERTE: a form of partial or fully paid leave mandated by employers. ** includes engineer, architect, license, undergraduate studies.

**Table 2:** Mixed methods table of recurring themes and sub-themes from qualitative interviews (*n* = 40).

Theme	# of Mentions	*n* (%) Female	Age Category *n* (%) *	Notable Quotes
**Change in Routine**				
*General change in routine*	20	10 (50)	a. 7 (35) b. 9 (45) c. 4 (20)	“I didn’t use video calls before, I was a little anti-video call and now I do 2–3 long video calls a day with friends, even group video calls. I talk to my friends that I’ve had since I was a kid and we stay in contact, have some wine, and share about our weeks.” —*Male, 54, Castilla-Leon* “As for friends, I’m always on my phone, I’m a person who likes to respond as soon as possible, so yea I have a lot of interactions with people and friends, especially on WhatsApp, we talk a lot. On Skype, yea I’ve done a few video calls and what’s more…they’re long video calls, 3 or 4 h, but above all I’m on WhatsApp and Instagram.” —*Male, 22, Navarra* “In normal circumstances I would never have dedicated this much time to WhatsApp, one thing is that you have relationships, the other is that you’re at home all day, so now yea, it’ is one more source of relationships, and when I open WhatsApp I practically have to be there for an hour and a half reading and responding, this is outrageous.” —*Male, 73, Basque Country* “Actually, I had deleted all of my social media accounts just before all of this happened, and now with everything I reactivated Instagram because if I didn’t, I wouldn’t see any of the things that are happening…” —*Male, 54, Castilla-Leon* (In response to being asked if there were any new activities that they have started that they did not do before COVID) “Above all it’s the use of social media, I didn’t use social media much before and now the recipes that I’m inventing I share with people, and this more than anything, the truth is that the only activity that I’m doing now that I didn’t do before…it’s trying to be in contact with more people, to know how one’s feeling, sharing how it is to be locked up.” —*Male, 24, Andalucía*
*A lot of time on the phone in general*	18	10 (55.6)	a. 6 (33.3) b. 8 (44.4) c. 4 (22.2)	
*A lot of time on phone/video calls*	14	10 (71.4)	a. 5 (35.7) b. 7 (50) c. 2 (14.3)	
*A lot of time on social media*	3	0 (0)	a. 2 (66.7) b. 0 (0) c. 1 (33.3)	
*More social media use compared to pre-COVID*	5	1 (20)	a. 1 (20) b. 4 (80) c. 0 (0)	
*Less social media use compared to pre-COVID*	1	0 (0)	a. 0 (0) b. 0 (0) c. 1 (100)	
*More contact than pre-COVID*	11	6 (54.5)	a. 5 (45.5) b. 5 (45.5) c. 1 (9.1)	
*No change in routine*	2	2 (100)	a. 1 (50) b. 1 (50) c. 0 (0)	
**Social Support**				
*Contact with family*	53	31 (58.5)	a. 17 (32.1) b. 28 (52.8) c. 8 (15.1)	“One thing that I’ve seen is that many mutual support networks are being generated amongst the neighbors, there are various WhatsApp groups that I am invited to, but direct contact with them, I’ve never had.” —*Female, 29, Catalonia* “We share information with our friends from all parts of the world; with those from Italy, my friends from Chile and Oceania. They’re countries very far away, and we want to know how everything is going there. Nurse the relationships that we already have, talk with those we didn’t talk to before, communicate with people from other countries and see how they’re doing, and inform them as well. Share experiences. They keep me informed on what’s going on in Chile and I inform them on what’s going on here.” —*Female, 36–55 (exact age not specified), Catalonia* “…I tell my daughter on WhatsApp to send the song of survival, it’s like it comforts me, it fills me and I hold on to that spirit, that soon this will all pass and we will be together and things will go back to how they were before, and this is the little ray of light that we have now.” —*Female, 44, Madrid* “…Yes I get together virtually with my friends, two or three times a week and I drink my beer with them through the phone, but it’s nothing like being in person, and I believe that in a city like ours, Mediterranean, physical contact for us is so important, that through a screen, well it’s just not the same.” —*Female, 30, Madrid*
*Contact with friends*	62	34 (54.8)	a. 24 (38.7) b. 33 (53.2) c. 5 (8.1)	
*Contact with neighbors*	2	2 (100)	a. 1 (50) b. 1 (50) c. 0 (0)	
*International contact*	6	6 (100)	a. 1 (16.7) b. 4 (66.7) c. 1 (16.7)	
*Communicate for social support*	22	15 (68.2)	a. 7 (31.8) b. 12 (54.5) c. 3 (13.6)	
*Virtual contact not the same as social support*	7	4 (57.1)	a. 4 (57.1) b. 3 (42.9) c. 0 (0)	
**Seeing Faces/Being Face-to-Face**	13	3 (23.1)	a. 3 (23.1) b. 7 (53.8) c. 3 (23.1)	“Well yes I am missing human contact, being face-to-face. I am someone who speaks to the face and I need to have people in front of me. Never, since I was young, have I spoken on the phone, it’s always face-to-face, for me to have made a phone call it had to have been something really urgent or important, I have always enjoyed speaking face-to-face. I’ve just discovered video calls now, about 3 weeks ago, it was something I always rejected, I didn’t like them or talking on the phone. I like physical contact.” —*Male, 54, Castilla-Leon*
**Media for News**				
*General media source for news*	13	7 (53.8)	a. 2 (15.4) b. 5 (38.5) c. 6 (46.2)	“…I’m not on social media much, well, I have WhatsApp which I use with a group of friends but I’m not much, I’ve never been, before or now, and now even less because they bombard you with false news and things like this…” —*Male, 62, Madrid* “We watch the news midday and that’s it, we see a little bit of what’s happening, how the death figures are, now we’re seeing they’re going down a little, and that’s it. I do look at social media for news too, you look at Twitter and see a little about what’s happening and when I see everyone getting heated, above all on Twitter the people get so heated at nothing.” —*Male, 31, Valencia* “The TV, no I don’t watch it, but if I feel like watching sometimes I watch the news. But then I see all the problems, I don’t believe what they’re saying, then what I see makes me anxious…I don’t watch it.” —*Female, 41, Extremadura* “WhatsApp is a thing that can get a little excessive, everything you receive, since sometimes you’ll see that they’re not reliable things, and really I’m not one to participate and distribute things that aren’t very secure or that don’t seem to me to be truthful.” —*Male, 73, Basque Country* “I haven’t looked at the news in many days because it makes me so mad, it makes me so mad to see the disgusting politicians that we have, it makes me nervous.” —*Male, 43, Aragon* “…I have another uncle who is very delicate and is hospitalized (with COVID)…nevertheless I get many very difficult stories from close friends through WhatsApp groups.” —*Female, 50, Galicia* “…I spend a lot of time watching television and on social media, and really, I do not recommend it because they’re really toxic, completely toxic, no?” —*Female, 50, Catalonia*
*WhatsApp for news*	4	0 (0)	a. 1 (25) b. 0 (0) c. 3 (75)	
*YouTube for news*	1	0 (0)	a. 0 (0) b. 0 (0) c. 1 (100)	
*Internet for news*	3	2 (66.7)	a. 0 (0) b. 2 (66.7) c. 1 (33.3)	
*Social media for news*	6	4 (66.7)	a. 2 (20) b. 2 (20) c. 2 (20)	
*Television for news*	5	2 (40)	a. 0 (0) b. 2 (40) c. 3 (60)	
*Fake/dangerous news on social media/WhatsApp*	7	4 (57.1)	a. 1 (14.3) b. 3 (42.9) c. 3 (42.9)	
*Frustration/anxiety from news*	6	3 (50)	a. 1 (16.7) b. 3 (50) c. 2 (33.3)	
*Media to stay informed*	4	3 (75)	a. 1 (25) b. 2 (50) c. 1 (25)	
*Communicate to share COVID news and updates*	6	4 (66.7)	a. 2 (33.3) b. 3 (50) c. 1 (16.7)	
*Do not like to read/see news*	4	2 (50)	a. 1 (25) b. 2 (50) c. 1 (25)	
**Media for Hobbies/Healthier Living**				
*General mentions of media use for hobbies*	11	7 (63.6)	a. 4 (36.4) b. 6 (54.5) c. 1 (9.1)	“…All of the classes on Instagram, I’m doing a lot of yoga and pilates which was something that I had never done until now. I went to the gym but I’ve never exercised in this way, nor utilized Instagram for it, and the truth is that it’s going really well for me.” —*Female, 30, Madrid* “We spend a lot of the time in the kitchen cooking, and we’re following cooks on Instagram that we like a lot and we cook together.” —*Male, 35, La Rioja* “I thought that at home, there way nothing I’d be to do, I thought I couldn’t exercise if it wasn’t with a tape, and I found a video online of an American girl who does exercises that I can do for my physical conditions and my joints and such and I do the exercises. I believe everything has an alternative way.” —*Female, 44, La Rioja* “Every day we do exercise with a girl we follow on Instagram, and we do exercises with her. For cooking as well, we cook online with a chef from here. There’s also concerts, that you watch from home, on Instagram and we do that as well.” —*Male, 35, La Rioja*
*Social media for cooking*	5	1 (20)	a. 2 (40) b. 3 (60) c. 0 (0)	
*Social media for exercise*	7	6 (85.7)	a. 2 (28.6) b. 4 (57.1) c. 1 (14.3)	
*Social media for learning*	4	3 (75)	a. 2 (50) b. 2 (50) c. 0 (0)	
**Virtual Events/Hangouts**				
*Virtual events/hangouts in general*	10	2 (20)	a. 6 (60) b. 3 (30) c. 1 (10)	“I cook a lot, I never cooked or ate well before, the truth is that I always ate healthy but it was really basic food and now maybe I make more elaborate meals but I learned everything on video calls with friends where they showed me how to make food.” —*Male, 54, Castilla-Leon* “We used to get together to have some wine and some tapas or a sandwich with my parents or with friends, and now we can’t do that, we’ve substituted all of it virtually.” —*Female, 35, Aragon* “Before, I would get ready and go out midday for a bite, well now I do the same but I have my food on video calls. Now I do that, I talk to some people, and in a group that we’ve formed we send photos of the food we’re eating.” —*Male, 54, Castilla-Leon* “…I talk with my friends almost every day, the video calls are great, but when it’s been three weeks doing it, well, the joke is that we’re already starting to get tired of it.” —*Female, 30, Madrid* “We’re doing a lot of online activities, so for example, we have a group of board games, escape rooms and puzzles, and we’re organizing all of these activities to do with the distance, so three times a week, we pick an activity and we get together virtually with the whole team and play. It’s all going really well for us and then later we also try to play on-line with our friends.” —*Female, 30, Madrid*
*Virtual celebrations*	1	1 (100)	a. 0 (0) b. 1 (100) c. 0 (0)	
*Virtual drinks*	10	4 (40)	a. 6 (60) b. 4 (40) c. 0 (0)	
*Video calls during meals*	7	2 (28.6)	a. 3 (42.9) b. 4 (57.1) c. 0 (0)	
*Video fatigue*	5	4 (80)	a. 2 (40) b. 2 (40) c. 1 (20)	
**Pandemic Relief**				
*Media use for pandemic relief*	6	5 (83.3)	a. 1 (16.7) b. 4 (66.7) c. 1 (16.7)	“I enjoy the little things now, like a video call with friends makes me super happy. So now I find joy with what little I have, it’s like we have to hold on to what we have because if we don’t, we’ll die.” —*Female, 21, Galicia* “Well at first it is a bit boring, you have a computer, telephone, WhatsApp, emails, and all of that which helps you cope, but in the end it is a bit tiring because it’s every day and it’s a little boring. Later you feel like you need to breathe air, and even though you can go out on your balcony, but breathe air from taking a walk, take a walk by the river, I don’t know, what we did regularly before, it’s not a lot of things but that’s what you miss, you feel like you’re a little locked up. But oh well, we don’t lack anything, we have all of the possibilities to speak to family with video calls. In reality we’re privileged, I’ve heard my parents’ stories from the war, and that was a hard time, but we’re in a transitioning situation, but I hope by June it will all be over and little by little we can go back to our normal lives.” —*Male, 73, Basque Country* “Well, to see each other, you know, video calls and then the typical WhatsApp, you know, if it’s humorous its better, avoid the politics, try to avoid the statistics on the news, that’s what television is for, but above all the funny memes because if you’re not laughing, then…” —*Female, 41, Extremadura…*
*Tech use for relaxation*	2	2 (100)	a. 1 (50) b. 1 (50) c. 0 (0)	
*Privileged for technology during pandemic*	2	1 (50)	a. 0 (0) b. 1 (50) c. 1 (50)	
*Memes*	3	2 (66.7)	a. 0 (0) b. 2 (66.7) c. 1 (33.3)	
*Comic relief from memes*	2	1 (50)	a. 0 (0) b. 1 (50) c. 1 (50)	

* Age category: a = 18–35, b = 36–55, c ≥ 55.

**Table 3:** Demographic characteristics of Wave 1 (*n* = 6789) and Wave 2 (*n* = 3500) survey participants.

	Wave 1	Wave 2
Variable	*n* (%)	*n* (%)
Age		
18–34	1802 (26.5)	739 (21.1)
35–60	3535 (52.1)	1880 (53.7)
61+	1452 (21.4)	881 (25.2)
Gender		
Female	3534 (52.2)	1720 (49.1)
Other	4 (0.1)	44 (1.3)
Civil Status		
Single	2715 (40.0)	978 (27.9)
Married	3251 (47.9)	2133 (60.9)
Widowed	166 (2.4)	86 (2.5)
Legally separated	148 (2.2)	53 (1.5)
Divorced	509 (7.5)	250 (7.1)
Education *		
No studies	26 (0.4)	14 (0.4)
Primary studies	304 (4.5)	208 (5.9)
Elemental Bachelor’s	878 (12.9)	380 (10.9)
FP1 or 2	1444 (21.3)	794 (22.7)
Superior Bachelor’s	1222 (18.0)	595 (17.0)
Technical	1032 (15.2)	519 (14.8)
Advanced degree	1833 (27.0)	968 (27.7)
Other	50 (0.7)	22 (0.6)
Occupational Status Before Confinement		
Public sector	1175 (17.3)	634 (18.1)
Private sector	2398 (35.3)	1123 (32.1)
Entrepreneur	399 (5.9)	193 (5.5)
On leave (sick, vacation, maternity)	119 (1.8)	50 (1.4)
Not working	895 (13.2)	472 (13.5)
Retired	971 (14.3)	623 (17.8)
Student	303 (4.5)	105 (3.0)
Homemaker	288 (4.2)	149 (4.3)
Permanent incapacity for work	109 (1.6)	66 (1.9)
Other economic inactivity	132 (1.9)	39 (1.1)
On ERTE **	-	46 (1.3)
Pandemic effect on work		
Lost job permanently	118 (1.7)	197 (5.6)
Lost job temporarily	861 (12.7)	165 (4.7)
Maintained my job	2993 (44.1)	1891 (54.0)
Maintained situation of inactive employment	-	558 (15.9)
COVID-19 Risk Group ***		
Yes	2251 (33.2)	-
Had COVID-19 Symptoms		
No	5934 (87.4)	-
Yes but did not take a diagnostic test	803 (11.8)	-
Diagnosed with COVID-19, not hospitalized	40 (0.6)	-
Hospitalized with COVID-19	12 (0.2)	-
Sought psychological help during confinement		
Yes	238 (3.5)	-

* Elemental Bachelor’s = Secondary studies; FP1 or 2 = Non-University Level technical/vocational degree; Superior Bachelor’s = Higher Secondary studies; Technical = University first stage; Advanced Degree = University second stage/Master’s degree; Other = mostly doctorate degrees. ** ERTE: a form of partial or fully paid leave mandated by employers. *** 60+ years old, chronic illness, immunosuppressed, pregnant, among others.

**Table 4:** Changes in social media use from April 2020 (*n* = 6789) to April 2021 (*n* = 3500).

	April 2020 *		April 2021 **	
Changes in Social Media Use	*n*	%	*n*	%
Increased a lot	2122	31.3	616	17.6
Increased a little	2553	37.6	871	24.9
The same	1735	25.6	1643	46.9
Decreased a little	114	1.7	94	2.7
Decreased a lot	62	0.9	62	1.8
N/a	203	3	190	5.4

* How much have your social media habits changed during confinement? ** Since summer 2020, how have your social media habits changed?

**Table 5:** Wave 1 associations between social media use and gender and age.

Variable	Social Media Use *n* (% of Total Category)			χ2
	Decreased	No Change	Increased	
Gender **				107.53 *
Male (*n* = 3126)	87 (2.8)	976 (31.2)	2063 (66)	
Female (*n* = 3460)	89 (2.6)	759 (21.9)	2612 (75.5)	
Age **				178.04 *
18–34 (*n* = 1778)	51 (2.9)	375 (21.1)	1352 (76)	
35–60 (*n* = 3428)	104 (3)	884 (25.8)	2440 (71.2)	
61+ (*n* = 1380)	21 (1.5)	476 (34.5)	883 (64)	

* *p* < 0.05. ** participants who responded ‘not applicable’ are excluded from total numbers.

**Table 6:** Wave 1 perceived social support and loneliness by social media use and Chi-square associations during confinement in April 2020 (*n* = 6789).

Variable and Level of Support	Social Media Use *n* (%)			χ2
	Decreased	No Change	Increased	
Physical or Medical problems				146.10 *
little to no help	42 (0.62)	6789 (4.4)	679(10.0)	
some help	51 (0.75)	492 (7.2)	1305 (19.2)	
a lot of help	83 (1.2)	945 (13.9)	2691 (39.6)	
Psychological problems				143.70 *
little to no help	60 (0.88)	624 (6.2)	1005 (14.8)	
some help	46 (0.68)	532 (7.8)	1458 (21.5)	
a lot of help	70 (1.0)	779 (11.5)	2212 (32.6)	
Need for social support				187.80 *
little to no help	43 (0.63)	246 (3.6)	499 (7.4)	
some help	40 (0.59)	403 (5.9)	981 (14.4)	
a lot of help	93 (1.4)	1086 (16.0)	3195 (47.1)	
Need to buy food or supplies				118.60 *
little to no help	33 (0.49)	253 (3.7)	566 (8.3)	
some help	38 (0.56)	377 (5.5)	859 (12.7)	
a lot of help	105 (1.5)	1105 (16.3)	3250 (47.9)	
Home infrastructure problems				94.79 *
little to no help	55 (0.81)	363 (5.3)	923 (13.6)	
some help	53 (0.78)	525 (7.7)	1489 (21.9)	
a lot of help	68 (1.0)	847 (12.5)	2263 (33.3)	
Need to run errands				134.10 *
little to no help	39 (0.57)	345 (5.1)	778 (11.5)	
some help	57 (0.83)	523 (7.7)	1423 (21.0)	
a lot of help	80 (1.2)	867 (12.8)	2474 (36.4)	
Feelings of Loneliness				288.04 *
Decreased	26 (0.38)	85 (1.3)	62 (0.91)	
No change	125 (1.8)	1162 (17.1)	435 (6.4)	
Increased	357 (5.3)	2461 (36.2)	1938 (28.5)	

* *p* < 0.05.

**Table 7:** Wave 2 Survey data: Social media use among individuals who did or did not have difficulty accessing important daily supplies during April 2021 (*n* = 3500).

	Social Media Use * n (%)			χ2
	Decreased	No Change	Increased	
Difficulties **				46.80 ****
Yes	30 (0.85)	297 (8.5)	355 (10.1)	
No	203 (5.8)	1346 (38.5)	1132 (32.3)	
(If yes) Stress Level ***				81.24 ****
Not very stressful	9 (1.2)	85 (11.8)	89 (12.3)	
Somewhat stressful	8 (1.1)	101 (14)	122 (16.9)	
Very Stressful	13 (1.8)	111 (15.4)	144 (19.9)	

* Since last summer, how have your social media habits changed?. ** In the last 30 days, have you had difficulty accessing important daily supplies (e.g., sanitary supplies, food, clothes, water, medication). *** If yes, how stressful was the difficulty in access to these supplies? *n* = 722. **** *p* < 0.05.

## Data Availability

The data presented in this study are available on request from the corresponding author.

## Appendix A

**Table A1:** Description of subject areas for the semi-structured interview questions.

Block Number	Main Concept
Block 1	Employment information *(e.g., status, changes due to the pandemic, teleworking)*
Block 2	Housing characteristics *(e.g., number of occupants, available spaces, reasons for exiting, protection measures inside and outside the home)*
Block 3	Perception of COVID-19 and lockdown *(e.g., perception of the future impact of the pandemic and lockdown in the personal sphere, impact to individual rights)*
Block 4	Basic habits *(e.g., changes in daily routine, eating, exercise, sleep schedule, substance use)*
Block 5	Conciliation *(e.g., effect of lockdown on family and friend relationships)*
Block 6	Emotional impact *(e.g., feelings about the lockdown, assimilation difficulties, possible signs of anxiety or depression, optimism, future impact of the pandemic on health, COVID-19 diagnoses in self or loved ones)*
Block 7	Coping strategies *(e.g., coping behaviors, use of social support, participation in solidarity actions)*
Block 8	Self-control *(e.g., ability to protect self during the pandemic)*
Block 9	Final assessment, additional questions or comments, best and worst aspects of lockdown
